# The effect of cognitive control and flexibility on perceptions of professional identity in teachers: the multimediating role of career adaptability, career engagement, and career optimism

**DOI:** 10.3389/fpsyg.2025.1716818

**Published:** 2026-02-09

**Authors:** Hazel Duru, Osman Söner

**Affiliations:** 1 Educational Sciences, Bursa Uludag Universitesi, Nilüfer, Türkiye; 2 Psychological Counseling and Guidance, Istanbul Sabahattin Zaim University, Istanbul, Türkiye

**Keywords:** career adaptability, career engagement, career optimism, cognitive control and flexibility, perception of professional identity

## Abstract

**Introduction:**

Teachers' professional identity has recently become a prominent focus in educational research, as it is closely related to teachers' effectiveness, motivation, and professional performance. Identifying the psychological factors that strengthen teachers' sense of professional identity is therefore critical, particularly in challenging professional contexts.

**Methods:**

This study examined the associations between cognitive control/flexibility and teachers' professional identity in Türkiye, with an emphasis on the sequential and multiple mediating roles of career optimism, career engagement, and career adaptability. The sample consisted of 372 teachers (175 women and 197 men). Data were analyzed using structural equation modeling and serial multiple mediation analysis, with the integrated use of SPSS, AMOS, and JASP software.

**Results:**

The findings revealed that career optimism, career adaptability, and career engagement functioned as significant serial and multiple mediators in the relationship between cognitive control/flexibility and professional identity. While cognitive control/flexibility showed limited direct effects, their influence on professional identity was transmitted primarily through career-related psychological resources. In addition, individual characteristics, particularly age, were found to influence these processes.

**Discussion:**

The results highlight the importance of fostering career development, motivation, and psychological flexibility in supporting teachers' professional identity. These findings offer practical implications for educational policy and teacher development programs, emphasizing the role of career-related resources in sustaining teachers' professional effectiveness and long-term career engagement.

## 1 Introduction

Teachers' professional identities are seen as a critical element that directly affects the quality of education systems and have therefore been intensively researched in recent years (Izadinia, 2013). Although there is no clear definition of identity, it has been emphasized that teachers should consider their professional identity as a resource for understanding themselves (Beijaard et al., 2004; Coldron and Smith, 1999). Professional identity provides a basis for teachers to develop themselves and engage in new learning, and it is a dynamic process that involves the reconciliation of personal and professional aspects (Beauchamp and Thomas, 2009; Pillen et al., 2013; Olsen, 2011; Rodgers and Scott, 2008). This process is influenced by teachers' personal characteristics, learning backgrounds, experiences, and professional contexts (Beauchamp and Thomas, 2009; Hong, 2010). By 2024, approximately 1.2 million people will be practicing the teaching profession in Türkiye (Republic of Türkiye, Ministry of National Education, 2024). However, despite being a critical profession, teaching faces numerous challenges. Indeed, research has revealed that only 26% of teachers feel valued professionally in society, salaries are below the OECD average, and class sizes are overcrowded (Demirel Yazici and Cemaloglu, 2022). Recent research has indicated that low salaries, the discrediting of the profession, and excessive student numbers are among the problems frequently mentioned by teachers (Iş and Atiş, 2024). Considering these studies, it appears that teachers as a professional group in Türkiye face many factors that can negatively impact their sense of professional identity. Based on this, the researchers tested the model they developed to identify the psychological factors that can positively predict teachers' professional identity perceptions and ensure that practices are carried out in this direction.

### 1.1 Cognitive control flexibility and professional identity perception

Essentially, cognitive flexibility refers to an individual's ability to switch between mental structures and strategies to adapt to changing environments and conditions (Ionescu, 2012; Laureiro-Martínez and Brusoni, 2018). The model developed by Gabrys et al. (2018) emphasizes that cognitive control/flexibility play a key role in individuals' ability to adapt to new events and situations. Cognitive control allows individuals to manage recurring negative emotions and thoughts, develop coping strategies, and make effective decisions. Research shows that individuals with high cognitive flexibility have stronger communication skills, more flexible career perceptions, and a greater sense of responsibility (Lan, 2023; Söner, 2023). In teaching, cognitive flexibility can be considered an important dimension of professional competencies (Kiliç and Demir, 2012). In addition to general competencies such as communication, collaboration, self-assessment, and professional development (Ministry of National Education, General Directorate of Secondary Education, 2017), teachers also need cognitive flexibility to adapt to their situations (Çuhadaroglu, 2013) effectively. At this point, cognitive flexibility can be considered an important element associated with the formation of teachers' professional identities. Professional identity includes self-perceptions and how others define that individual professionally (Danielewicz, 2001; Golombek and Johnson, 2017; Neary, 2014). By acting on these perceptions, individuals construct their identities (Oyserman et al., 2017), determine their goals and values (Ruohotie-Lyhty and Moate, 2016), and, thanks to their professional identity, they can lead more robust, compelling, and harmonious professional lives (Beijaard et al., 2004; Day et al., 2006). In this study, cognitive control and cognitive flexibility were treated as a unified construct. Although they are theoretically distinct, these two cognitive functions are closely interrelated in practice. To examine their integrated impact on teachers' professional identity, the “Cognitive Control and Flexibility Scale” was utilized, as it covers both structures and provides a composite total score. Consequently, cognitive control/flexibility were evaluated as a single dimension in the analyses to investigate how this combined cognitive capacity relates to career engagement and professional identity. Based on this, our research aimed to examine the role of cognitive control/flexibility, which may be associated with one of the individual aspects of professional identity, in teachers' perceptions of their professional identity. We hypothesized that cognitive control/flexibility could be a significant factor in predicting teachers' perception of their professional identity. Based on this, our first hypothesis is:

H1. *A positive and significant relationship exists between teachers' cognitive control/flexibility and their perception of their professional identity*.

### 1.2 Career optimism as a mediator

Career optimism is defined as individuals' expectation of achieving the best possible outcome in their careers, focusing on positive aspects of career development, and maintaining a positive outlook on career planning (Rottinghaus et al., 2005). Hennessey et al. (2008) defined this concept as individuals' confidence to meet job demands arising from variable and challenging work conditions. Career optimism is also a developable psychological state (Higgins et al., 2010) and encourages individuals to develop positive expectations regarding career outcomes (Yang et al., 2017). Individuals with high career optimism actively plan for their future, engage in career self-management more effectively (Ahmad and Bilal, 2023), evaluate new career opportunities more realistically (Kim et al., 2014), and address the events they encounter from positive perspectives (Santilli et al., 2017). In the literature, career optimism demonstrates positive relationships with many variables such as career adaptability (McLennan et al., 2017), career satisfaction (Haratsis et al., 2015), academic self-esteem (Kartol and Söner, 2022), and professional identity (Eva et al., 2020). Career optimism may be associated with teachers' perceptions of professional identity as a motivating psychological factor, playing a critical role in teachers facing challenges related to policies and work. Furthermore, because career optimism is a developable aspect (Higgins et al., 2010), it is anticipated that teachers' cognitive flexibility can increase this optimism. Indeed, there are limited studies in the literature supporting the relationship between optimism and cognitive flexibility (Dogan Lacin and Yalçin, 2019; Sapmaz and Dogan, 2013). The limited number of studies and their cross-sectional nature suggest that the directionality of these relationships may be reciprocal. As teachers with cognitive flexibility can generate alternatives in the face of various challenges and cope effectively, their optimism may be higher. This may indicate a reciprocal relationship between these two variables. Based on all these, our second hypothesis in this study is:

H2. *Career optimism mediates the relationship between teachers' cognitive control/flexibility and their perception of professional identity*.

### 1.3 Career engagement as a mediator

Career engagement refers to how individuals develop their careers through various proactive behaviors. In this context, career planning, exploration, and skill development are concrete examples of career engagement (Hirschi et al., 2014; Hirschi and Freund, 2014). Research has shown that career engagement is related to various factors, such as individuals' personality traits and cognitive abilities, and can mediate career-related outcomes (Hirschi and Jaensch, 2015). Cognitive control enables individuals to engage in planned activities to achieve their goals, while cognitive flexibility offers the opportunity to develop alternative solutions when faced with uncertainties and challenges. Therefore, teachers possessing these skills may show higher levels of career engagement by supporting their proactive career behaviors. Another factor directly linked to teachers' career engagement is career optimism. Career optimism allows individuals to develop positive expectations and see their careers as worth investing in. Optimistic individuals are more proactive in their career activities and more successful in achieving their goals (Perera and McIlveen, 2014; Söner et al., 2024). Career engagement has been found to be related to perceptions of professional identity. It has been emphasized that individuals with a strong career identity may exhibit higher levels of career engagement (Wilk and Moynihan, 2005), and research has revealed a strong link between professional identity and work engagement (Kim and Kang, 2017; Wu et al., 2022). In this context, teachers' active planning and career investment make it easier for them to see their profession as part of their identity and contribute to strengthening their sense of professional identity. Based on all these findings, the third and fourth hypotheses of the study propose that career engagement may mediate the relationship between teachers' cognitive flexibility and their perceptions of professional identity, as well as the relationship between their career optimism and their perceptions of professional identity. Based on these findings, our third and fourth hypotheses are as follows:

H3. *Career engagement mediates the relationship between teachers' cognitive control/flexibility and their perceptions of professional identity*.

H4. *Career engagement mediates the relationship between teachers' career optimism and their perceptions of professional identity*.

### 1.4 Career adaptability as a mediator

Career adaptability is a fundamental concept that explains individuals' adaptation processes when transitioning from school to work, from one job to another, or during career changes (Savickas and Porfeli, 2012). This construct, consisting of four dimensions, encompasses concern (awareness and expectations about the future), control (shaping oneself and the environment through efforts), curiosity (exploring future scenarios and possible selves), and confidence (belief in success despite challenges). A review of the literature reveals that career adaptability is positively correlated with variables such as hope and optimism (Buyukgoze-Kavas, 2016; Jiang, 2017), career awareness (Söner, 2025), career success (Zacher, 2014), career obstacles (Duru and Söner, 2025), and career engagement (Ochoco and Ty, 2022; Öztemel and Yildiz-Akyol, 2025). They suggested that career adaptability may mediate between protean career orientation and career optimism (Chui et al., 2022). Career engagement helps individuals solve the career challenges they face and, for teachers, enables them to become better prepared for changing circumstances through behaviors aimed at improving their profession. Furthermore, career optimism is closely related to career adaptability; it is considered an indicator of personal adaptability (Fang et al., 2018; Rudolph et al., 2017) and has been linked to individuals' resilience in professional life (Nguyen et al., 2016). Various studies confirm the relationship between career optimism and adaptability (Chui et al., 2022; Delle and Searle, 2022; McLennan et al., 2017). Teachers' perception of a hopeful future supports their engagement in planned behaviors (Concern), control beliefs (Control), trying alternative paths (Curiosity), and trusting despite challenges (Confidence). (Demirtaş and Kara 2022) have shown significant relationships between these constructs. Therefore, our study suggests that teachers' cognitive control/flexibility support their career adaptability. Furthermore, the relationship between perception of professional identity and career adaptability is important in the context of two meta-competences of career development (Hall, 2002). While studies generally suggest that professional identity predicts career adaptability (Liu et al., 2023; Qiao et al., 2022), some findings reveal that these two constructs interact (Negru-Subtirica et al., 2015). Therefore, teachers' career adaptability may be associated with a stronger perception of professional identity and contribute to a more meaningful internalization of their profession as a part of their identity. In our study, we propose that career adaptability is associated with professional identity perception. We believe that teachers with career adaptability will be more curious about their profession and more likely to explore and take responsibility. Teachers with career adaptability can more effectively cope with the challenges they face, evaluate their options, and make decisions. This, in turn, can enable teachers to identify with their profession and develop their perception of professional identity. Based on this, the final hypotheses of our study are as follows:

H5. *Teachers' career adaptability mediates their cognitive control/flexibility, and perception of professional identity*.

H6. *Teachers' career adaptability mediates their career optimism and their perception of professional identity*.

H7. *Teachers' career adaptability mediates their career engagement and their perception of professional identity*.

### 1.5 The present study

This study aims to make an original theoretical and methodological contribution to the educational psychology literature by proposing an integrated model to explain the cognitive and career-based psychological processes that related to teachers' perceptions of their professional identity. In professions that require emotional labor, such as teaching, the construction of this identity is influenced by career-related psychological variables and individual resources (Beauchamp and Thomas, 2009; Akkerman and Meijer, 2011). In this context, the study tested the effects of cognitive control/flexibility on teachers' perceptions of their professional identity, assuming they mediate transitional psychological constructs such as career optimism, proactive career engagement, and adaptability. This model, structured based on career construction theory (Savickas, 2005) and self-regulation theories, hypothesizes that individuals' psychological resources interact to shape their professional self-perception. This study demonstrates that, rather than the direct impact of cognitive control/flexibility in a teacher sample, these variables indirectly influence professional identity by activating positive career tendencies. Career optimism, in particular, is positioned as a central psychological transition point in this model, strengthening the perception of professional identity by influencing career engagement and adaptability (McLennan et al., 2017). Furthermore, it has been shown that career engagement, not only as a motivational tendency but also when it gains meaning in conjunction with an individual's psychological adaptability, can have an impact on professional identity (Nilforooshan and Salimi, 2016). By integrating the moderating effects of demographic factors, particularly age, on these processes into the model, the study offers a more comprehensive framework that considers contextual variables (Jisong, 2024). Therefore, the primary objective of this study is to test the predictive role of teachers' cognitive control/flexibility levels on their professional identity perceptions within a holistic model, through the sequentially interacting career psychology components.

## 2 Method

### 2.1 Research design

This study tested a chained multiple mediation model to explain the effects of cognitive control/flexibility on perceptions of professional identity. In the model, cognitive control/flexibility were the independent variables, and perception of professional identity was the dependent variable. Career adaptability, career optimism, and career engagement were modeled as mediating variables in this relationship. Direct and indirect effects between variables were assessed using serial multiple mediation analysis (Hayes, 2013). The model's adequacy was tested using structural equation modeling (SEM), and the significance levels of the relationships between variables were confirmed (Kline, 2016).

### 2.2 Participants' procedure

The study participants consisted of 382 teachers from different cities in Türkiye. Data collected for the study were analyzed after identifying outliers and conducting normality tests. Following Z-score analysis and data cleaning, data from 372 participants were included in the final analysis. Of the participating teachers, 175 (47.0%) were female and 197 (53.0%) were male. The average age of the participants was 37.26, and the average number of years working in the profession was 11.77. Regarding the distribution by institution, 35 participants (9.4%) worked in preschool education, 85 (22.8%) in primary schools, 97 (26.1%) in middle schools, 118 (31.7%) in high schools, and 37 (9.9%) in other public institutions such as Guidance and Research Centers and Public Education Centers. In terms of employment status, 166 teachers (44.6%) were employed as permanent staff, 106 (28.5%) as contract teachers, and 100 (26.9%) as part-time teachers.

### 2.3 Measurement

#### 2.3.1 Cognitive control and flexibility scale

The *Cognitive Control and Flexibility Scale*, developed by Gabrys et al. (2018) and adapted into Turkish by (Demirtaş 2019), was employed to assess teachers' levels of cognitive control/flexibility. In the Turkish adaptation study, a total of 640 participants were included across three stages: 47 participants (66% female, 34% male) for linguistic equivalence testing, 241 participants (65% female, 35% male) for construct validity and reliability analyses, and 352 participants for examining construct relations, internal consistency, and item validity. The 18-item scale consists of two theoretically meaningful subdimensions—*Cognitive Control over Emotions* and *Appraisal and Coping Flexibility*—which are conceptualized as closely interrelated components of a broader self-regulatory capacity rather than independent processes. Cognitive control over emotions reflects individuals' ability to regulate affective responses, whereas appraisal and coping flexibility represents the capacity to generate adaptive interpretations and coping strategies; together, these processes operate synergistically in adaptive functioning (Gabrys et al., 2018). Accordingly, in the present study, cognitive control and flexibility were modeled as a unified construct using the total scale score. This decision was guided by both theoretical considerations emphasizing the integrated nature of these processes and methodological considerations aimed at reducing model complexity and enhancing parameter stability within a chained multiple mediation framework. The original two-factor structure of the scale demonstrated good model fit in the Turkish adaptation (χ^2^/df = 2.63, NFI = 0.94, CFI = 0.96, GFI = 0.86, AGFI = 0.82, IFI = 0.96, SRMR = 0.07, RMSEA = 0.08), and internal consistency coefficients were reported as 0.85 for Cognitive Control over Emotions and 0.91 for Appraisal and Coping Flexibility (Demirtaş, 2019). In the current study, the overall internal consistency of the composite cognitive control/flexibility score was high (Cronbach's α =0.909), supporting its use as an integrated indicator of cognitive self-regulatory capacity.

#### 2.3.2 Professional identity perception scale

The “Professional Identity Perception Scale,” developed by Yıldız and Çetin (2020) to measure teachers' perceptions of professional identity, was used in this study, was created due to a comprehensive process using both qualitative and quantitative methods. During the scale development process, a 43-item item pool was created based on data from semi-structured interviews with 20 teachers from different career stages, based on Engeström (1987) activity theory. It was shaped with expert opinions and reduced to a 5-factor, 17-item structure through exploratory factor analysis (EFA). It was determined that the factors explained 65.92% of the total variance. Confirmatory factor analysis (CFA) results (χ^2^/df=1.930, GFI = 0.934, CFI = 0.956, NFI = 0.892, TLI = 0.946, RMSEA = 0.046) revealed that the model fit well. Validity studies for the scale were conducted within the scope of content, construct, convergent, and divergent validity; reliability was tested using Cronbach's alpha (ranging from 0.70 to 0.86 for subdimensions), Spearman-Brown, Gutman split-half test coefficients, and composite reliability values. The findings indicate that the scale can measure teachers' perceptions of professional identity validly and reliably. As a result of the reliability analysis we conducted with data from the “Perception of Professional Identity Scale” for this study, Cronbach's alpha (α) reliability coefficient was determined to be 0.833.

#### 2.3.3 Career optimism scale

The “Career Optimism Scale,” which was used in this study to assess participants‘ positive expectations about their careers, was based on a subdimension of the Career Futures Inventory (CFI) scale developed by Rottinghaus et al. (2005). It was adapted to Spanish by Garcia et al. (2015) and Turkish by (Kepir Savoly and Tuzgöl Dost 2021). The scale has a single-factor, 11-item structure that measures individuals' positive thoughts about their careers. According to confirmatory factor analysis (CFA) in the Turkish adaptation study, goodness of fit indices supporting the construct validity of the scale [RMSEA = 0.087, CFI = 0.97, NFI = 0.95, SRMR = 0.058, χ2(224) = 716.91, (CI = 415.76–577.66), χ2/df = 3.20] indicate a good level of model fit. The scale's internal consistency was assessed using Cronbach's alpha reliability coefficient, which was calculated as 0.88. Furthermore, item-total correlations were determined to range between 0.524 and 0.855. The reliability analysis we conducted using this study's “Career Optimism Scale” data yielded a Cronbach's alpha (α) reliability coefficient of 0.979.

#### 2.3.4 Career adaptability scale

The “Career Adaptability Scale – Short Form (CASSF),” which was first created by Maggiori et al. (2017) and translated into Turkish by Isik et al. (2018), is used in this study to evaluate the adaptation issues people face during the career growth process. The scale, which has 12 items divided into four subscales—anxiety, control, curiosity, and confidence—was created using Savickas's (2005) Career Construction Theory as a basis. According to the results of confirmatory factor analysis, the scale's four-factor structure offered a satisfactory degree of model fit (χ2/df = 2.13, GFI = 0.950, CFI = 0.966, TLI = 0.955, RMSEA = 0.059). For the subscales, the scale's internal consistency coefficients varied from 0.80 to 0.91. On the other hand, the total scale's Cronbach's alpha was determined to be 0.82. The reliability analysis that we carried out. For the subscales, the scale's internal consistency coefficients varied from 0.80 to 0.91. On the other hand, the total scale's Cronbach's alpha was determined to be 0.82. Cronbach's alpha (α) reliability coefficient was 0.920, according to the reliability analysis we performed using the “Career Adaptability Scale” data for this study.

#### 2.3.5 . Proactive career engagement scale

In this study, the “Proactive Career Engagement Scale” used to assess individuals' proactive behaviors toward their career development processes was developed by Hirschi et al. (2014), and its Turkish adaptation was carried out by Korkmaz et al. (2020). The scale measures individuals' active participation behaviors, such as career planning, skill acquisition, professional networking, and exploring career opportunities. 699 university students from all over Türkiye participated in the Turkish adaptation research, and confirmatory factor analysis (CFA) showed that the scale's single-factor, nine-item structure was maintained in line with its original form. The scale's model fit indices (χ^2^ = 118.12, sd = 24, CFI = 0.95, TLI = 0.93, RMSEA = 0.09, SRMR = 0.06) were determined to be at an acceptable level. Cronbach's alpha coefficient was 0.88, indicating internal consistency dependability; the 4-week test-retest correlation was stated to be 0.67. The scale's good internal consistency was also demonstrated by reliability assessments conducted for this study; Cronbach's alpha was determined to be 0.951. Cronbach's alpha (α) reliability was the result of the reliability analysis we performed using the “Proactive Career Engagement Scale” data for this study.

### 2.4 Ethical process

Participation in the study was entirely voluntary. All participants were informed about the purpose of the research, the procedures, confidentiality principles, and their right to withdraw from the study at any stage. Written informed consent was obtained from all participants. Since all participants were over 18, additional parental or legal guardian consent was not required. The research process was conducted per the ethical principles of the Declaration of Helsinki, ensuring confidentiality, anonymity, and voluntariness.

### Data analysis

The online survey form was sent to teachers working in different cities in Türkiye using voluntary participation forms. The average time required for participants to complete all surveys was 18 min. The online survey form was sent to 852 teachers, and responses were received from 382. This corresponds to a response rate of 44.8%, which is considered acceptable for voluntary online survey research conducted with teacher samples (Dillman et al., 2014; Nulty, 2008). After outliers were removed from the collected data, analyses were conducted with the remaining 372 data points. During the data analysis process, normality assumptions were tested, and first, skewness-kurtosis values and Z-score distribution were examined. Morgan et al. (2004) stated that a skewness-kurtosis value between −1 and +1 indicates a normal distribution. The values for perception of professional identity (Skew.: −1.104, Curt.: −1.195), career engagement (Skew.: −0.591, Curt.: −0.059), cognitive control/flexibility (Skew.: −0.460, Curt.: −0.602), career adaptability (Skew.: −0.890, Curt.: −0.150), and career optimism (Skew.: −1.423, Curt.: 1.423) also supported the assumption of normality. Subsequently, the scatter of the Z-score distribution also confirmed the normality assumption. To assess potential common-method bias arising from self-report and single-wave data collection, Harman's single-factor test was conducted. All measurement items were entered into an unrotated exploratory factor analysis, and the results indicated that the first factor accounted for less than 50% of the total variance, suggesting that common-method variance was not a serious concern in the present study (Podsakoff et al., 2003). Regarding sample size adequacy, methodological guidelines for structural equation modeling indicate that sample sizes above 200 are sufficient for stable parameter estimation in models of moderate complexity (Kline, 2016), and that a minimum ratio of 5–10 participants per estimated parameter is recommended (Bentler and Chou, 1987). Given the number of free parameters estimated in the present chained multiple mediation model, the final sample size of 372 participants exceeds these recommended thresholds. Furthermore, the use of bias-corrected bootstrapping with 5,000 resamples enhances the statistical power and robustness of indirect effect estimates in complex mediation models (Hayes, 2013). Furthermore, a parameterized structural equation model requires adherence to the operational definitions of theoretical variables and specification of the expected functional forms for causal effects (Kline, 2016). Structural equation modeling (SEM) with direct and indirect effects has been tested to identify multiple mediating roles in the chain. SPSS 29.0 and AMOS 29.0 were used for analyses. The JAMOVI program was also used to determine the relationship between variables with 95% confidence intervals, and JASP was used to visualize these relationships using Bayesian network analysis. To determine the fit of the developed model, *x*^2^/df, RMSEA, CFI, GFI, and IFI values were examined, and the model was found to be a good fit.

## 3 Findings

The relationship coefficients and confidence intervals between teachers' career adaptability, career optimism, career engagement, cognitive control/flexibility, and professional identity perception were analyzed with the JAMOVI program, and the results are presented in Table 1.

**Table 1:** Descriptive values and correlational relationships for variables.

**Descriptive statistic**						**Correlation values**			
**Variables**	***M***	**df**	α	**Skew**.	**Curt**.	**Correlations with professional identity perception**		**Confidence intervals**	
						***r***	***p***	**95% CI upper**	**95% CI lower**
Perception of professional identity	62.20	18.31	0.833	−1.104	1.195				
Proactive career engagement	33.02	8.02	0.935	−0.591	−0.059	0.350	< 0.01	0.436	0.257
Cognitive control and flexibility	94.96	16.58	0.909	−0.460	0.602	0.289	< 0.01	0.380	0.193
Career adaptability	53.02	6.45	0.920	−0.890	0.150	0.401	< 0.01	0.483	0.312
Career optimism	93.67	18.32	0.979	−1.126	1.423	0.416	< 0.01	0.497	0.329

According to the information in Table 1, the relationships between perception of professional identity and other variables were examined using Pearson's correlation coefficient. According to the findings, a positive and moderately significant relationship was found between career engagement and perception of professional identity (*r* = 0.350, *p* < 0.01, 95% CI [0.257, 0.436]). Similarly, a positive and significant relationship was found between the cognitive control/flexibility variable and perception of professional identity (*r* = 0.289, *p* < 0.01, 95% CI [0.193, 0.380]). The career adaptability variable was found to be positive and moderately related to perception of professional identity (*r* = 0.401, *p* < 0.01, 95% CI [0.312, 0.483]). Furthermore, the relationship between career optimism and perception of professional identity stands out as the strongest, and this relationship is positive and significant (*r* = 0.416, *p* < 0.01, 95% CI [0.329, 0.497]). These results indicate that an increase in individuals' career-related attitudes and skills also strengthens their perception of professional identity.

**Figure 1:**
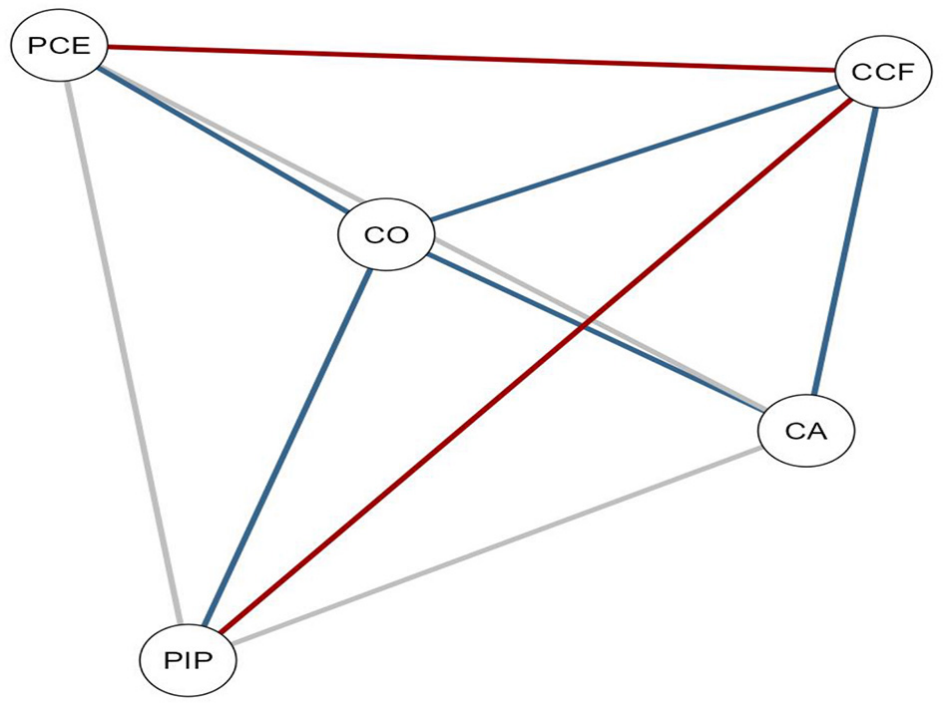
Bayesian network analysis of variables. PIP, professional identity perception; CO, career optimism; PCE, proactive career engagement; CA, career adaptability; CCF, cognitive control and flexibility.

A Bayesian network analysis was conducted to examine the conditional dependencies and probabilistic relationships between variables. In the Bayesian analysis, positive conditional relationships between variables are shown with blue lines, and negative conditional relationships are shown with red lines, identifying variables that support or constrain perception of professional identity. The analysis process was conducted using JASP software, and the resulting network structure and visual outputs are presented in Figure 1.

The visualization of the Bayesian network analysis presented in Figure 1 reveals how the impact of cognitive control/flexibility (CCF) on perception of professional identity (PIP) in teachers is structured in a multilevel manner through career engagement (PCE), career optimism (CO), and career adaptability (CA). The positive conditional dependencies shown by the blue lines in the network indicate powerful connections between CCF, CO, and CA, and these constructs are positively related to PIP. CO is a central transition point between CCF and PIP and a mediator between PCE and CA. On the other hand, the red lines between PCE and CCF and PIP indicate negative conditional relationships, indicating that excessive proactive career engagement can limit both flexibility capacity and perception of professional identity.

After determining the relationship between perception of professional identity and other variables, a model was constructed using AMOS, and regression analysis was conducted to determine the predictive value of each variable. The regression weights and significance levels of these analyses are presented in Table 2.

**Table 2:** Regression weights.

**Dependent Variable**	**Direction**	**Independent Variable**	**Estimate**	**S.E**.	**C.R**.	**P**
CO	< –	CCF	0.371	0.044	8.401	< 0.05
PCE	< –	CCF	0.078	0.035	2.205	< 0.05
CA	< –	CCF	0.082	0.014	5.818	< 0.05
PCE	< –	CO	0.423	0.050	8.383	< 0.05
CA	< –	PCE	0.058	0.023	2.467	< 0.05
CA	< –	CO	0.086	0.021	4.155	< 0.05
PIP	< –	CO	0.042	0.014	3.027	< 0.05
PIP	< –	PCE	−0.001	0.016	−0.038	>0.05
PIP	< –	CA	0.172	0.044	3.866	< 0.05

PIP, professional identity perception; CO, career optimism; PCE, proactive career engagement; CA, career adaptability; CCF, cognitive control and flexibility.

According to the regression analysis results presented in Table 2, the cognitive control/flexibility variable plays a positive and significant predictive role on both career optimism (β = 0.371, *p* < 0.05), career engagement (β = 0.078, *p* < 0.05), and career adaptability (β = 0.082, *p* < 0.05). Similarly, career optimism significantly predicts both career engagement (β = 0.423, *p* < 0.05) and career adaptability (β = 0.086, *p* < 0.05), while career engagement contributes to career adaptability (β = 0.058, *p* < 0.05). When the direct effects on professional identity perception were examined, career optimism (β = 0.042, *p* < 0.05) and career adaptability (β = 0.172, *p* < 0.05) stood out as significant predictors, while the effect of career engagement was adverse but not significant (β = −0.001, *p* > 0.05). These results suggest that teachers' professional identity development is shaped more by psychological adaptation and positive expectations, while proactive career engagement is not a direct factor in this process.

**Figure 2:**
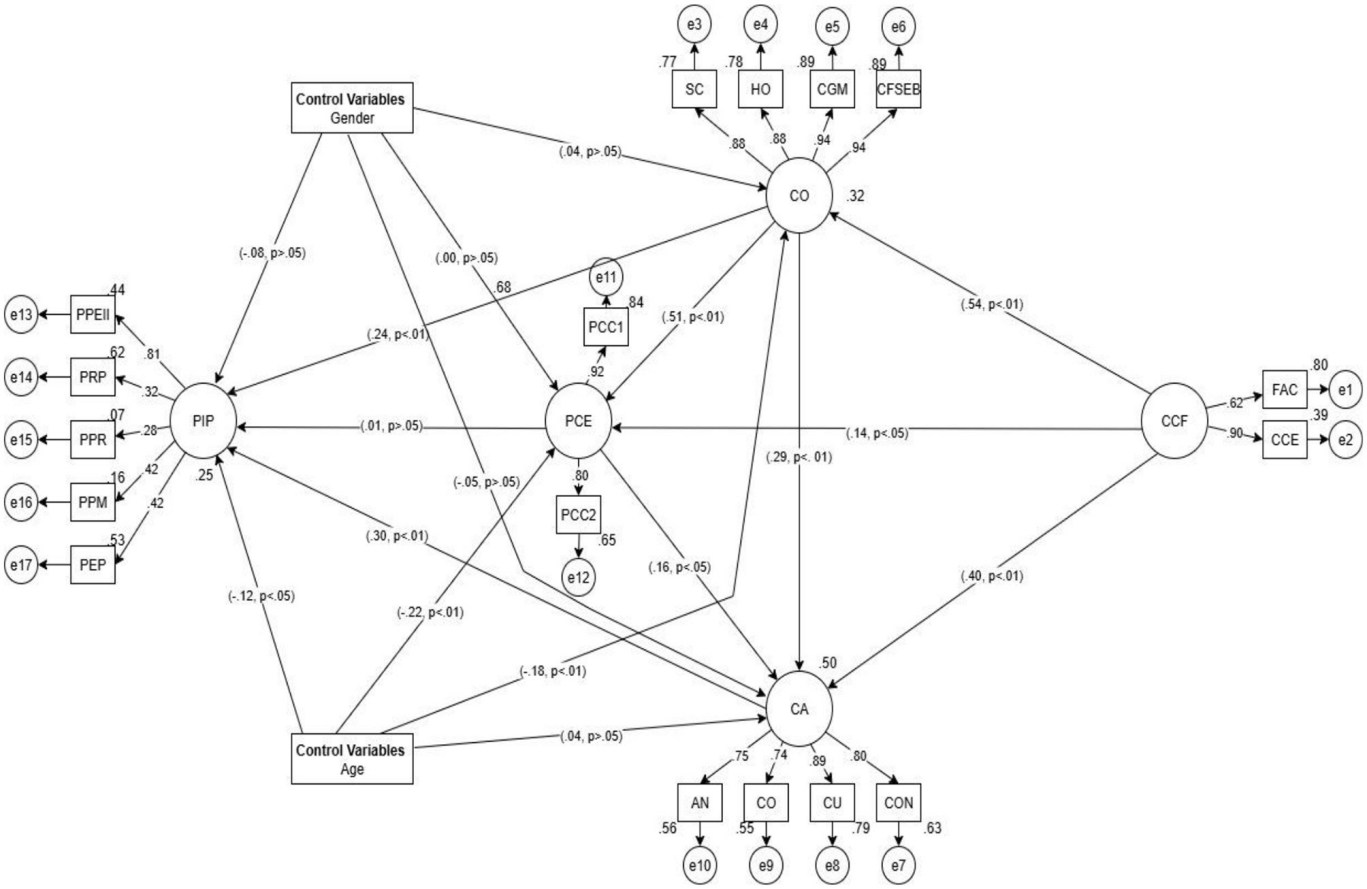
Serial mediation model. PIP, professional identity perception; PPEII, perception of professional educational and instructional interaction; PRP, professional role perception; PPR, perception of professional respectability; PPM, perception of professional motivation; PEP, perception of experiential pedagogy; CO, career optimism; SC, self confidence; HO, hope; CGM, career goals motivation; CFSEB, career future self-efficacy beliefs; PCE, proactive career engagement; PAC1 (Item 1„ 2., 3., 4., 5.) PAC 2 (Item 6., 7., 8., 9.); CA: AN, anxiety; CO, control; CU, curiosity; CON, confidence; CCF, cognitive control and flexibility; FAC, flexibility to assess and cope; CCE, cognitive control over emotions.

Based on these findings, the findings of the structural equation modeling conducted to determine the chained multiple mediating role of career adaptability, career optimism, and career engagement in the effect of cognitive control/flexibility on professional identity perception are presented in Figure 2.

As seen in Figure 2, regarding the effect of cognitive control/flexibility on perception of professional identity, firstly, CCF has a strong and direct effect on career optimism (CO; β = 0.54, *p* < 0.01), indicating that individuals with more flexibility and higher levels of control hold positive expectations for their careers. CO, in turn, significantly predicts both career engagement (PCE; β = 0.29, *p* < 0.01) and career adaptability (CA; β = 0.40, *p* < 0.01), thus forming the second and third links of the mediation chain. Career engagement also directly and significantly affects career adaptability (β = 0.16, *p* < 0.05), indicating that committed individuals adapt their career paths more. The most significant indirect effect within this chain structure is the CCF → CO → CA → PIP orientation. In terms of direct effects, CO (β = 0.24, *p* < 0.01) and especially CA (β = 0.30, *p* < 0.01) significantly predicted the perception of professional identity. In contrast, the direct effect of PCE on PIP was insignificant (β = 0.01, *p* > 0.05). These findings indicate that cognitive control/flexibility capacity do not alone determine professional identity, but instead exert indirect and multilevel effects through positive attitudes toward one's career and levels of psychological adjustment. Age, one of the control variables included in the model, has a significant and negative effect on both career engagement (β = −0.22, *p* < 0.01) and career adaptability (β = −0.18, *p* < 0.01), suggesting that the levels of these two variables may decrease with increasing age. On the other hand, gender had no significant effect on any variable (all *p* > 0.05) and did not contribute to the structural model.

Overall, the model suggests that career optimism, career adaptability, and career engagement play a multi-mediating role in the impact of cognitive control/flexibility on perception of professional identity, and that this process may be influenced by individual factors such as age. The model fit values for the paths in the diagram of the developed model are presented in Table 3.

**Table 3:** Fit values of the structural equation model tested.

**Goodness of Fit Indices**	**Obtained variables**	**Good fit**	**Acceptable fit**	**References**
*x*^2^/sd	3.820	≤ 3	≤ 4–5	Carmines and McIver, 1981
RMSEA	0.087	≤ 0.05	0.06–0.08	Browne and Cudeck, 1992
SRMR	0.067	≤ 0.05	0.06–0.08	Hu and Bentler, 1999
CFI	0.906	≥0.95	0.90–0.94	Kline, 2016
GFI	0.870	≥0.95	0.90–0.94	Joreskog and Sorbom, 1984
IFI	0.906	≥0.95	0.90–0.94	McDonald and Marsh, 1990

As shown in Table 3, the structural equation model for the multiple mediating effects of career optimism, career adaptability, and career engagement on the impact of cognitive control/flexibility on perception of professional identity was statistically significant and met the criteria for fit. The coefficients for the direct and indirect effects determined by the multiple mediating effects, with a 95% confidence interval and 5,000 bootstrap loadings, are presented in Table 4.

**Table 4:** The bootstrapping coefficients and confidence intervals regarding the serial multiple mediation analysis.

**Parameter**									**Bootstrap coefficient^a^**	**Lower^b^**	**Upper^b^**	***p***
**Direct effects**												
CCF	–>				CO				0.371	0.279	0.485	0.000
CCF	–>				PCE				0.078	0.007	0.158	0.030
CO	–>				PCE				0.423	0.313	0.552	0.000
CCF	–>				CA				0.082	0.054	0.124	0.000
PCE	–>				CA				0.058	0.004	0.123	0.037
CO	–>				CA				0.086	0.036	0.143	0.003
CO	–>				PIP				0.042	0.007	0.085	0.019
PCE	–>				PIP				−0.001	−0.031	0.038	0.968
CA	–>				PIP				0.172	0.056	0.295	0.005
**Indirect Effect**												
CCF	–>	CO	–>	PCE	–>	CA	–>	PIP	0.009	0.001	0.021	0.030
CCF			–>	CO	–>	PCE	–>	PIP	0.000	−0.005	0.006	0.970
CCF			–>	CO	–>	CA	–>	PIP	0.005	0.002	0.013	0.014
CCF			–>		CO	–>		PIP	0.016	0.003	0.033	0.018
CCF			–>		PCE	–>		PIP	0.000	−0.003	0.003	0.930
CCF			–>		CA	–>		PIP	0.014	0.004	0.030	0.004

a. Bootstrap coefficients are based on 5,000 resamples. b. Lower and upper values represent the 95% bias-corrected bootstrap confidence intervals.

In the bootstrap analysis, the direct effects of CCF on career optimism (CO; β = 0.371, *p* < 0.001), career engagement (PCE; β = 0.078, *p* = 0.030), and career adaptability (CA; β = 0.082, *p* < 0.001) were found to be significant. Furthermore, CO directly significantly predicted both career engagement (β = 0.423, *p* < 0.001) and perception of professional identity (β = 0.042, *p* = 0.019). CA was also influenced by both CO and PCE (β = 0.086, *p* = 0.003 and β = 0.058, *p* = 0.037) and stood out as a direct and strong predictor of CCA (β = 0.172, *p* = 0.005). On the other hand, the direct effect of PCE on MKA was insignificant (β = −0.001, *p* = 0.968), indicating that engagement alone did not affect MKA. Regarding the indirect effects, five separate mediation paths were tested. The first and longest path, CCF → CO → PCE → CA → MKA, was found to be significant (β = 0.009, 95% CI [0.001, 0.021], *p* = 0.030), representing the most substantial chain mediation effect in the model. Second, the CCF → CO → CA → MKA path was also found to be significant (β = 0.005, *p* = 0.014), indicating that optimism contributes to MKA by supporting career adaptability. Third, the CCF → CO → MKA path was also significant (β = 0.016, *p* = 0.018), confirming the direct effect of CO on MKA. The fourth path, CCF → CO → PCE → MKA, was not significant (β = 0.000, *p* = 0.970), indicating that PCE does not support the transition from CO to MKA. Fifth and finally, the CCF → PCE → MKA path was also insignificant (β = 0.000, *p* = 0.930), suggesting that PCE alone does not indirectly contribute to MKA. Consequently, cognitive control/flexibility appear to significantly influence perception of professional identity, not directly but indirectly, particularly through chained pathways established through variables such as career optimism and adaptability.

## 4 Discussion

Our study identified factors associated with teachers' perceptions of their professional identity. Based on this, we considered teachers' professional identity perceptions, critical in their career development, cognitive control/flexibility, optimism, adaptability, and engagement. We also revealed the relationships between these variables using multiple mediation analyses. We confirmed all seven hypotheses we tested in the context of the study. Our first finding from the study is a positive and significant relationship between teachers' cognitive control/flexibility and their perceptions of professional identity (H1). Studies on the relationship between cognitive control/flexibility and their perceptions of professional identity are pretty limited in the literature. In their study, Galletta et al. (2024) found no significant relationship between cognitive control/flexibility and professional identity. Indeed, Kiliç et al. (2024) found a positive and significant relationship between cognitive flexibility and professional autonomy in their study. In this context, teachers with cognitive control/flexibility may be able to generate more effective alternative solutions to the challenges they face as they build their professional identities. They may also be able to adapt more easily to the innovations occurring in their rapidly changing professional lives. This may contribute to a more positive perception of teachers' professional identity.

The second finding from the research is that teachers' career optimism plays a mediating role between their cognitive control/flexibility and their perception of professional identity (H2). A review of the literature found no research examining these three variables together. However, there are also results similar to those in our study. (Demirtaş 2020) and (Peker-Akman 2024) found a positive and significant relationship between optimism and cognitive flexibility in their studies. In their study, (Yildiz-Akyol and Boyaci 2020) found a positive and significant relationship between career optimism and cognitive flexibility. However, no studies have examined career optimism and perception of professional identity together. Teachers with cognitive control/flexibility can approach the obstacles they encounter from different cognitive frameworks. This may give teachers a more optimistic perspective on their professional development. Furthermore, teachers with a positive perspective on their professional development may perceive themselves as more open to professional development and reinforce their positive beliefs about their professional identity. Teachers with career optimism may feel more competent in facing the obstacles, which may support their sense of belonging to their profession. Career optimism may be positively associated with teachers' perceptions of professional identity when these factors are considered together.

Our third finding from the study is that teachers' career engagement mediates between their cognitive control/flexibility and their perception of professional identity (H3). While no studies have examined all three variables in the literature, there are also results parallel to our research. Zhang and Guo (2023) found a positive and significant relationship between work engagement and professional identity in their study with teachers. Wu et al. (2022) found a positive and significant relationship between nurses' professional identity, work engagement, and career success. Kim and Kang (2017) found a similarly significant relationship between workers' career identity and work engagement. However, no studies have examined career engagement and cognitive control/flexibility in the literature. Results from the Structural Equation Modeling (SEM) indicate that cognitive control/flexibility is a variable that enhances teachers' career engagement. This finding aligns with theoretical expectations suggesting that cognitively flexible individuals can better adapt to professional demands and engage with their careers more meaningfully. However, the negative relationship identified between cognitive control/flexibility and career engagement in the Bayesian network analysis suggests that this interaction is neither linear nor unidirectional. In this context, exceptionally high levels of cognitive control/flexibility may lead teachers to adopt a more critical and inquisitive stance toward their professional roles. Teachers with superior cognitive control/flexibility might evaluate alternative roles rather than internalizing the teaching role as a single, dominant identity. As cognitive control/flexibility increases, teachers' commitment to their profession may evolve from an absolute attachment into a more conditional, questioning, and situational form of engagement. This highlights the necessity of evaluating the divergent relationships observed between SEM and Bayesian analyses as complementary insights. In this study, the direct effect of career engagement on professional identity was not significant. This suggests that career engagement may not uniformly strengthen professional identity across all contexts. Engagement may support professional identity development during early career stages. In contrast, high levels of engagement in later stages-without supporting psychological resources such as adaptability, flexibility, and optimism-may contribute to burnout or identity conflicts. Indeed, in their study, (Salmela-Aro and Upadyaya 2018) found that economic problems are associated with burnout symptoms during the early career stage, whereas in the late career stage, caregiving demands are linked to both increased burnout and decreased work engagement. Furthermore, career engagement is a multidimensional construct, including emotional, cognitive, and behavioral components. Future research could examine these sub-dimensions separately, along with career stages and teaching environments, to provide a more nuanced understanding. Finally, in the Turkish context, structural and environmental factors-such as appointment anxieties and economic challenges-may influence professional identity. The fourth finding from the study is that teachers' career engagement plays a mediating role between their career optimism and perceptions of professional identity (H4). A literature review reveals that, although no studies have examined these three variables together, some yield similar results. Krishnaveti et al. (2025) found a positive and significant relationship between career optimism, career adaptability, and career engagement, and that career optimism predicts career engagement. Teachers with an optimistic perspective on their careers may approach their profession with more hope, have greater confidence in their potential for career advancement, and develop greater engagement in their profession. Teachers who experience career engagement, on the other hand, may be more committed to their professional roles and, consequently, develop a stronger sense of identity.

Our fifth finding from the study is that teachers' career adaptability mediates between their cognitive control/flexibility and their perception of professional identity (H5). There is also research in the literature supporting this conclusion. Özen and Mete (2022) determined a positive and significant relationship between cognitive control/flexibility and career adaptability in healthcare workers. (Demirtaş and Kara 2022) also reached the same conclusion in their study. Yan et al. (2024) revealed that professional identity and career adaptability sub-dimensions have significant relationships. Haibo et al. (2018) also indicated a significant relationship between employees' career identity and adaptability. Teachers with cognitive control/flexibility may be better prepared for potential uncertainties that may arise in their careers. They may also adapt more easily to new situations and possess problem-solving skills. Teachers with career adaptability may also perceive themselves as having more control over their profession and internalize their teaching role. Consequently, they may have a positive perception of their professional identity. The sixth finding from the study is that teachers' career adaptability mediates between their career optimism and perception of professional identity (H6). Studies parallel our research findings. Chui et al. (2022), Delle and Searle (2022), McLennan et al. (2017), and Newman et al. (2022) found a positive and significant relationship between career optimism and career adaptability. Teachers' positive expectations about their careers may be related to a higher sense of control and curiosity regarding their careers. Teachers with career optimism may be more prepared and adaptable toward their careers. Teachers with career adaptability, in turn, may establish a more effective and healthy connection with their profession as they perceive themselves as able to manage uncertainties regarding their career futures. This may serve as an important resource influencing their perception of professional identity. The final finding from the study is that teachers' career adaptability plays a mediating role between their career engagement and perception of professional identity (H7). Ayvaz and Elhatip (2025), Yang et al. (2019), Öztemel and Yildiz-Akyol (2025), and Çarkit (2022) have demonstrated that career adaptability has a positive and significant relationship with career engagement. Teachers with career engagement may cope more effectively with the challenges they face in their profession. They may also be more willing to adapt to their careers. Individuals with adaptability may also identify more easily with their profession. Teachers' sense of control over their careers increases so that they can clearly define their professional roles.

## 5 Limitations and future directions

Our research has some limitations. We used a cross-sectional design. It should be noted that while the mediation effects were modeled statistically, the cross-sectional design of this study does not establish temporal precedence among the variables. Therefore, the observed pathways represent statistical associations rather than a chronological sequence. Consequently, we can only comment on the correlational level rather than determining cause-and-effect relationships. Future research could be conducted longitudinally. The reliance on self-report measures may introduce common method bias; however, statistical controls were applied to mitigate this potential issue. Furthermore, our study focused on individual psychological factors that influence the perception of professional identity. Future research could focus on contextual factors. Furthermore, our study considered several factors that could cognitively influence teachers' perception of professional identity. Future studies could also focus on emotional factors such as intelligence and hope. Our results are from a Turkish sample. Different factors may influence the perception of professional identity in different countries and cultures. Future studies could utilize experimental designs to further examine factors associated with teachers' perceptions of professional identity. Psychoeducation programs could include activities to increase teachers' career adaptability, psychological control, flexibility, and optimism. Group guidance could be conducted to raise teachers' awareness of their professional identity.

In addition, the findings of this study indicate that while several path coefficients are statistically significant, certain indirect effects—specifically the chained mediation pathway (CCF → CO → CA → PIP; β = 0.009) exhibit small effect sizes. From a statistical perspective, such a decrease in coefficients is anticipated in sequential mediation models, as the total effect is partitioned across multiple intervening variables. Although these minor effects might appear to offer limited immediate impact, their practical significance lies in the cumulative and long-term development of professional identity. In the context of teacher education and professional development, interventions targeting cognitive functions and psychological resources should not be viewed as “quick fixes.” Instead, they should be integrated into teacher training programs as sustainable and long-term modules. For instance, regular psychoeducational programs such as “Cognitive Reframing,” “Career Future Orientations,” and “Establishing Career Goals” can provide small but steady gains in teachers' adaptability and optimism. Over the course of a career, these incremental gains can significantly contribute to the strengthening of professional identity and increase vocational persistence.

## 6 Conclusion

This study examined how cognitive control/flexibility relate to teachers' perceptions of professional identity through the mediating roles of career optimism, career engagement, and career adaptability. The findings highlight that professional identity is not only shaped by external conditions but also by teachers' internal cognitive and career-related resources. Specifically, career optimism and adaptability emerged as the most potent mediators, suggesting that teachers' positive expectations for their careers and ability to adapt to challenges are significantly linked to a stronger professional identity. In contrast, proactive career engagement showed weaker and indirect effects, indicating that engagement alone may not directly enhance identity unless supported by optimism and adaptability. The results emphasize the importance of fostering psychological resources—such as flexibility, optimism, and adaptability—in teacher education and professional development programs. Strengthening these internal resources may help teachers maintain a resilient professional identity. despite structural challenges, ensuring greater effectiveness and commitment to their careers.

## Data Availability

The original contributions presented in the study are included in the article/Supplementary material, further inquiries can be directed to the corresponding author.
