# Correction: The effect of cognitive control and flexibility on perceptions of professional identity in teachers: the multimediating role of career adaptability, career engagement, and career optimism

**DOI:** 10.3389/fpsyg.2026.1807734

**Published:** 2026-03-02

**Authors:** Hazel Duru, Osman Söner

**Affiliations:** 1 Educational Sciences, Bursa Uludag Universitesi, Nilüfer, Türkiye; 2 Psychological Counseling and Guidance, Istanbul Sabahattin Zaim University, Istanbul, Türkiye

**Keywords:** career adaptability, career engagement, career optimism, cognitive control and flexibility, perception of professional identity

Yıldız and Çetin (2020) was not cited in the article. The citation has now been inserted in the section Method, Measurement Subsection, *2.3.2 Professional identity perception scale*, Paragraph 1 and should read:

“The “Professional Identity Perception Scale,” developed by Yıldız and Çetin (2020) to measure teachers' perceptions of professional identity, was used in this study.”

The original version of this article has been updated.
